# Epidemiology of circulating human influenza viruses from the Democratic Republic of Congo, 2015

**DOI:** 10.1371/journal.pone.0203995

**Published:** 2018-09-28

**Authors:** Hugo Kavunga-Membo, Edith Nkwembe, Edgar Simulundu, Stomy Karhemere, Pélagie Babakazo, Léonie Manya, Joelle Kabamba, Emile Okitolonda, Steve Ahuka-Mundeke, Jean Jacques Muyembe

**Affiliations:** 1 Département de Virologie, Institut National de Recherche Biomédicale (INRB), République Démocratique du Congo, RDC; 2 Department of Disease Control, School of Veterinary Medicine, University of Zambia, Lusaka, Zambia; 3 Ecole de Santé Publique, Université de Kinshasa (UNIKIN), Kinshasa, RDC; 4 Direction de lutte contre les Maladies, Ministère de la santé Publique, Kinshasa, RDC; The University of Hong Kong, CHINA

## Abstract

**Introduction:**

The establishment of the influenza sentinel surveillance system in Kinshasa, Bas Congo, Maniema, Katanga, and Kasai Provinces allowed generation of important data on the molecular epidemiology of human influenza viruses circulating in the Democratic Republic of Congo (DRC). However, some challenges still exist, including the need for extending the influenza surveillance to more provinces. This study describes the pattern of influenza virus circulating in DRC during 2015.

**Methodology:**

Nasopharyngeal swabs were collected from January to December 2015 from outpatients with influenza-like illness (ILI) and in all hospitalized patients with Severe Acute Respiratory Infection (SARI). Molecular analysis was done to determine influenza type and subtype at the National Reference Laboratory (NRL) in Kinshasa using real time reverse transcription-polymerase chain reaction (rRT-PCR). Analysis of antiviral resistance by enzyme inhibition assay and nucleotide sequencing was performed by the Collaborating center in the USA (CDC, Atlanta).

**Results:**

Out of 2,376 nasopharyngeal swabs collected from patients, 218 (9.1%) were positive for influenza virus. Among the positive samples, 149 were characterized as influenza virus type A (Flu A), 67 as type B (Flu B) and 2 mixed infections (Flu A and B). Flu A subtypes detected were H3N2 and H1N1. The Yamagata strain of Flu B was detected among patients in the country. Individuals aged between 5 and 14 years accounted for the largest age group affected by influenza virus. All influenza viruses detected were found to be sensitive to antiviral drugs such as oseltamivar, zanamivir, peramivir and laninamivar.

**Conclusion:**

The present study documented the possible involvement of both circulation of Flu A and B viruses in human respiratory infection in certain DRC provinces during 2015. This study emphasises the need to extend the influenza surveillance to other provinces for a better understanding of the epidemiology of influenza in DRC. It is envisioned that such a system would lead to improved disease control and patient management.

## Introduction

Influenza is a respiratory infection caused by influenza viruses [1]. It spreads rapidly among susceptible individuals, particularly during seasonal epidemics or pandemics and imposes a considerable economic burden attributed to increased hospitalizations among others [1]. Human influenza viruses are members of the *Orthomyxoviridae* family. In humans, only influenza A and B viruses (Flu A and B) are of epidemiological interest [2]. In Africa, for many years, influenza epidemiology was mainly described in countries with temperate climates like South Africa and Morocco [3, 4] Presently, the situation appears to be changing as considerable data in tropical countries such as the Democratic Republic of Congo (DRC), Kenya and Zambia has been reported [5, 6, 7]. On the African continent, influenza causes severe illness and deaths in both temperate and tropical settings [8, 9]. Identification and characterization of circulating influenza viruses is essential to detect the emergence of antigenic drift variants causing influenza epidemics. The detection of antiviral resistance and identification of novel A strains with the potential to cause an influenza pandemic are also needed [10]. Thus, influenza surveillance provides a basis for selection of the virus strains to be included in the annual formulation of influenza vaccines [11, 12].

**Fig 1:**
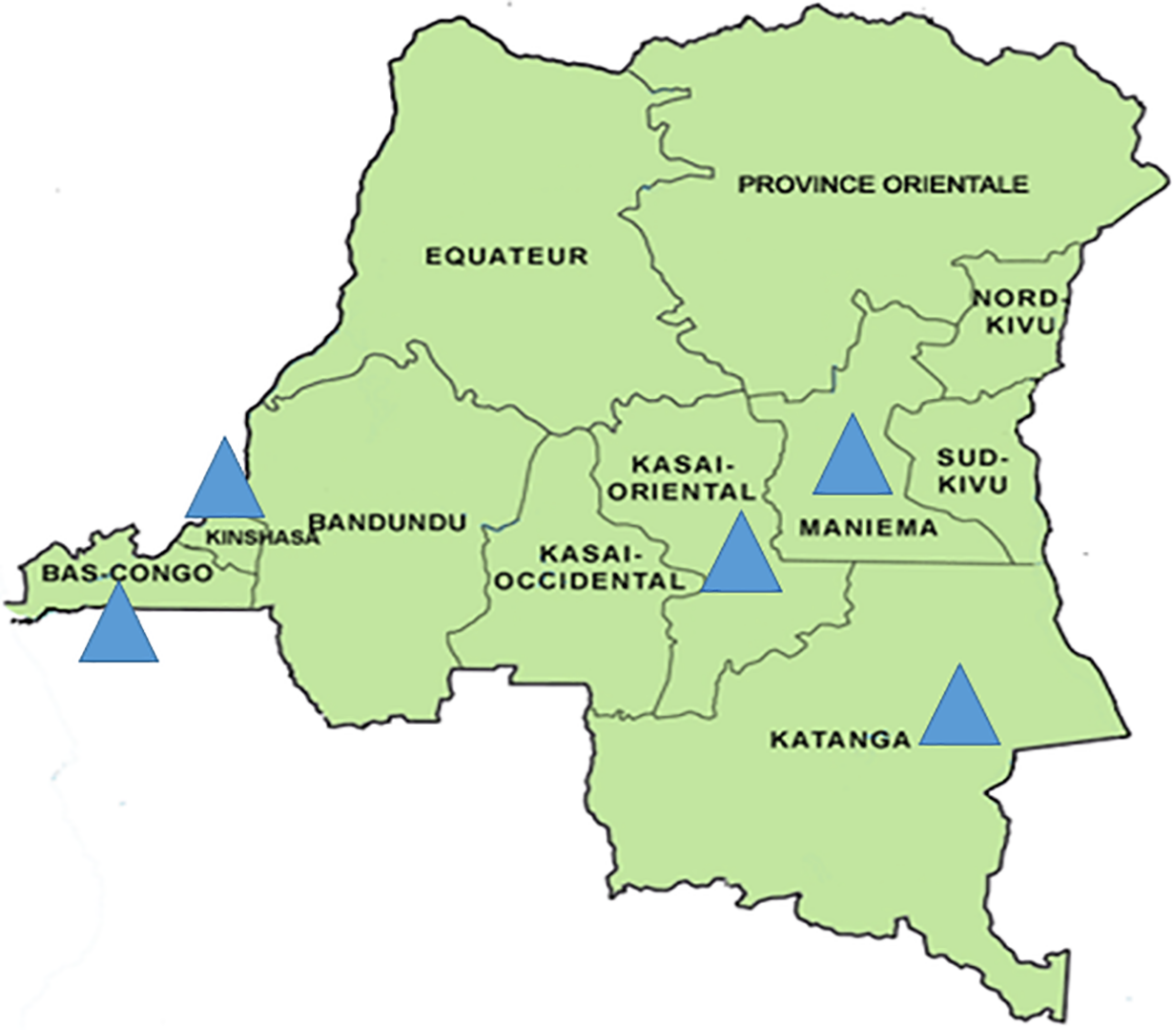
Map of Democratic Republic of Congo, the blue triangle represent provinces where flu surveillance is conducted.

The Influenza Sentinel Surveillance System was established in the DRC in 2008 following the pandemic threat posed by the Asian-origin H5N1 highly pathogenic avian influenza virus. The surveillance was established in 11 urban and rural sentinel sites in five of the 11 provinces of the country (Fig **1**). Those sites were selected on the basis of higher accessibility and affordability to patients, higher medical staff qualifications, and adequate specimen storage capacity. The surveillance system serves to monitor antiviral resistance, detect or identify novel influenza strains capable of causing a pandemic; to determine the epidemiology of influenza and other viral respiratory diseases; to characterize and monitor trends in disease and deaths from Severe Acute Respiratory Infection (SARI); to determine the proportions of influenza cases confirmed among patients hospitalized for SARI, to identify Influenza-like Illness (ILI) in outpatient, and to collect data with a view of understanding the disease burden in the country. This study was aimed at describing the pattern of influenza viruses circulating in DRC during 2015.

## Materials and methods

### Ethics statement

The influenza sentinel surveillance protocol was adapted from World Health Organization (WHO) guidelines with support from the national influenza surveillance program at the DRC Ministry of Health. This protocol was implemented as part of routine public health surveillance by the Ministry of Health and was therefore considered a service and not subject to human subjects review. However, some of the authors had access to identifying informations of the patients who participated in the surveillance program.

### Sample collection

Throat and/or nasal swabs obtained from patients with clinical evidence of ILI or SARI were collected using a cryovial which contained 3ml of viral transport medium and kept in the fridge (4–8°C) at the sentinel site, until they were packaged and transported on ice packs to The National Influenza Reference Laboratory (NIRL) in Kinshasa where they were aliquoted in tree different eppendorf tubes and kept frozen at −80°C before processing by real-time reverse-transcription polymerase chain reaction (rRT-PCR) assay. The NIRL, located at the Institut National de Recherche Biomédicale (INRB) is the only laboratory in DRC capable to perform influenza diagnostic by (rRT-PCR) and it has met the standards of the External Quality Assessment Program (EQAP) administered by WHO.

### RNA extraction and amplification

RNA was extracted from 140 μL obtained from an aliquot of 1 ml (from one eppendorf tube) using the QIAmp Viral RNA mini kit (Qiagen) and amplified using AgPath One-Step rRT-PCR for influenza virus typing (Ambion, Applied Biosystems) with the ABI 7500 Fast PCR Systems (Life Technologies). Samples were first tested for influenza virus type A and B by rRT-PCR, followed by rRT-PCR subtyping for H1N1, H3N2, H5N1 and H7N9 in samples that were positive for Flu A while those positive to Flu B were tested for B victoria and B yamagata.

### Assay for neuraminidase inhibitor susceptibility

Neuraminidase activity was measured using the fluorescent substrate, 2'- (4-methylumbelliferyl)-α-D-N-acetylneuraminic acid (MUNANA; Sigma) [13]. Briefly, 15 μl of virus was incubated with 30 μl of 100 μM MUNANA in 32.5 mM MES buffer pH 6.5 containing 4 mM CaCl_2_ for 1 hr at 37°C. The reaction was stopped by addition of 150 μl 0.14 M NaOH in 83% Ethanol and fluorescence of the released 4-methylumbelliferone was measured at excitation and emission wavelengths of 365 nm and 450 nm, respectively. The activity of each virus sample was titrated, by assaying serial twofold dilutions, and virus suspensions were adjusted to equivalent Neuraminidase activities, which fell in the linear portion of the activity curve. Each virus was preincubated for 30 minutes at 37°C with oseltamivar, zanamivir, peramivir and laninamivar at final concentrations of 5 μM-0.05pM, in serial 10-fold dilutions, Neuraminidase activity measured and the drug concentration that inhibited 50% of the neuraminidase activity (IC_50_) was determined [14].

### Sequence analysis

Isolates from DRC were sent to the collaborating center in USA (CDC, Atlanta) for sequencing and genetic characterization as previously described by Zhou *et al* and Shepard *et al* [15, 16]. Multiple sequence alignments were carried out for each data set using the CLC Main Workbench 5.7.2 software. A phylogenetic tree was inferred from each resulting nt sequence alignment by the Maximum-likelihood (ML) algorithm implemented in the MEGA version 7.7.1 software under the best substitution model (model having the lowest Bayesian Information Criterion), transition/transversion (Ts/Tv) ratio and ML base composition estimated from the empirical dataset [17, 18, 19]. For the tree topology, only trees from distance based analysis, requiring lesser space, are presented. Distance based phylograms were reconstructed by the neighbor-joining method with the Kimura two-parameter method for computing evolutionary distances for genetic distance determination and pairwise deletion for gaps [19].

## Results

### Molecular detection of influenza virus types and subtypes

**Fig 2:**
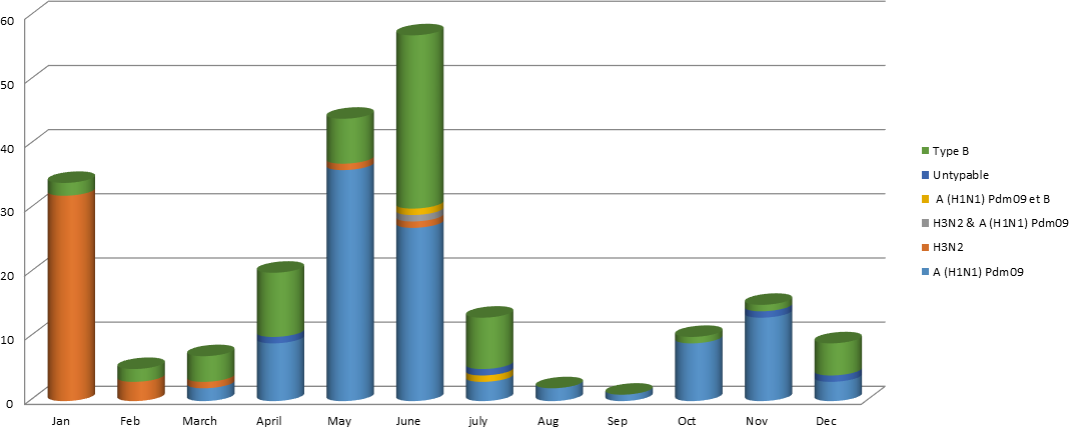
Frequency of type and subtype influenza virus reported by month during 2015.

During the study period, a total of 2,376 nasopharyngeal swabs were collected from 11 sentinel sites and analysed by the DRC surveillance system team. Of the samples analysed, rRT-PCR assay revealed that 218 samples were positive for influenza virus, of which 149 were characterized as Flu A, 67 as Flu B and two mixed infections (Flu A and B) (Table 1). The Flu A and B viruses were subjected to more detailed characterization by rRT-PCR, which indicated that among the 149 Flu A detected: 107 were classified as H1N1, 38 as H3N2 and 4 samples were untypeable while all isolated Flu B were determined as belonging to the B/Yamagata lineage. It was also noted that the incidence of Flu A (H3N2) was remarkably higher in January 2015 and decreased quickly, then disappeared in the second semester of the year 2015 (Fig 2).

**Table 1:** Distribution of Flu type by age group in 2015.

Flu type	Age group	0–4 years	5–14 years	15–24 years	25–40 years	>40 years	Total
Type A		42 (7.6%)	65 (9%)	14 (3.9%)	17 (4.5%)	11 (2.8%)	149 (6.2%)
Type B		20 (3.6%)	26 (3.6%)	8 (2.2%)	9 (2.4%)	4 (1%)	67 (2.8%)
Type A&B		2 (0.3%)	0 (0%)	0 (0%)	0 (0%)	0 (0%)	2 (0.9%)
Negative		484 (88.32%)	624 (87.2%)	331 (93.7%)	345 (92.9%)	374 (96.1%)	2158 (90.8%)
Total		548 (100%)	715 (100%)	353 (100%)	371 (100%)	389 (100%)	**2376 (100%)**

The present study revealed that the largest group of individuals affected by the disease was aged between 5 and 14 years, followed by those from 0 to 5 years (Table 1). During 2015, both Flu A and B co-circulated among human population, in the same province (Bas Congo, Kinshasa, Katanga) at same periods (Fig 2).

### Phylogenetic analysis of A (H1N1) pdm09, A (H3N2) and B (Yamagata)

**Fig 3:**
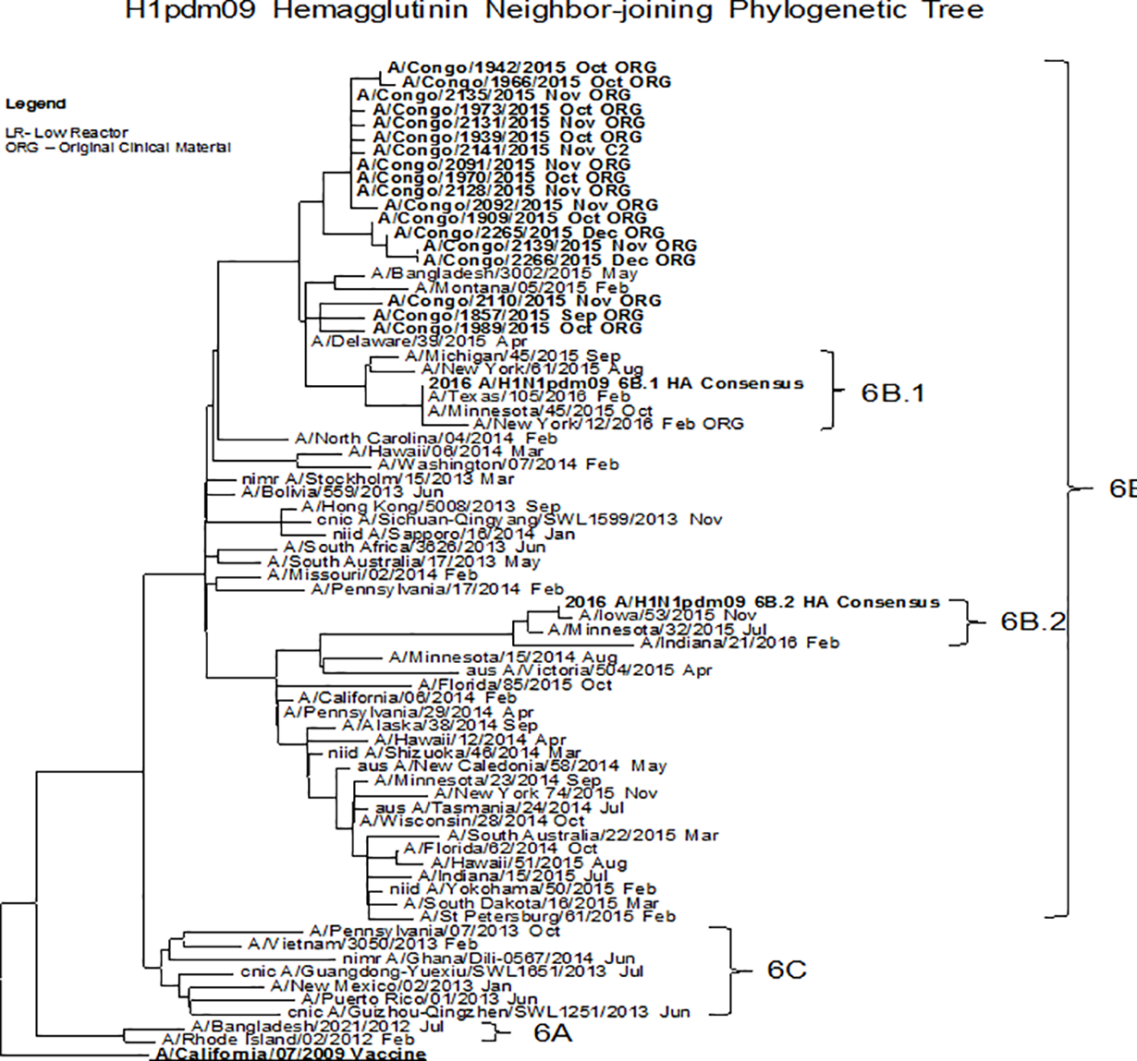
Representative hemagglutin Neighbor-joining phylogenetic tree of A (H1N1) pdm09 viruses collected in 2015. The A/California/07/2009 (Underlined, bold) was used as reference in TREESUB. The consensus HA tree and the transcribed aminoacid substitutions were visualized in fig Tree. The scalebar represents the average number of nucleotide substitutions per site. Genetic groups 6B, 6C, 6A are depicted in the tree. DRC isolates (bold) clustered with viruses from genetic groups 6B.

**Fig 4:**
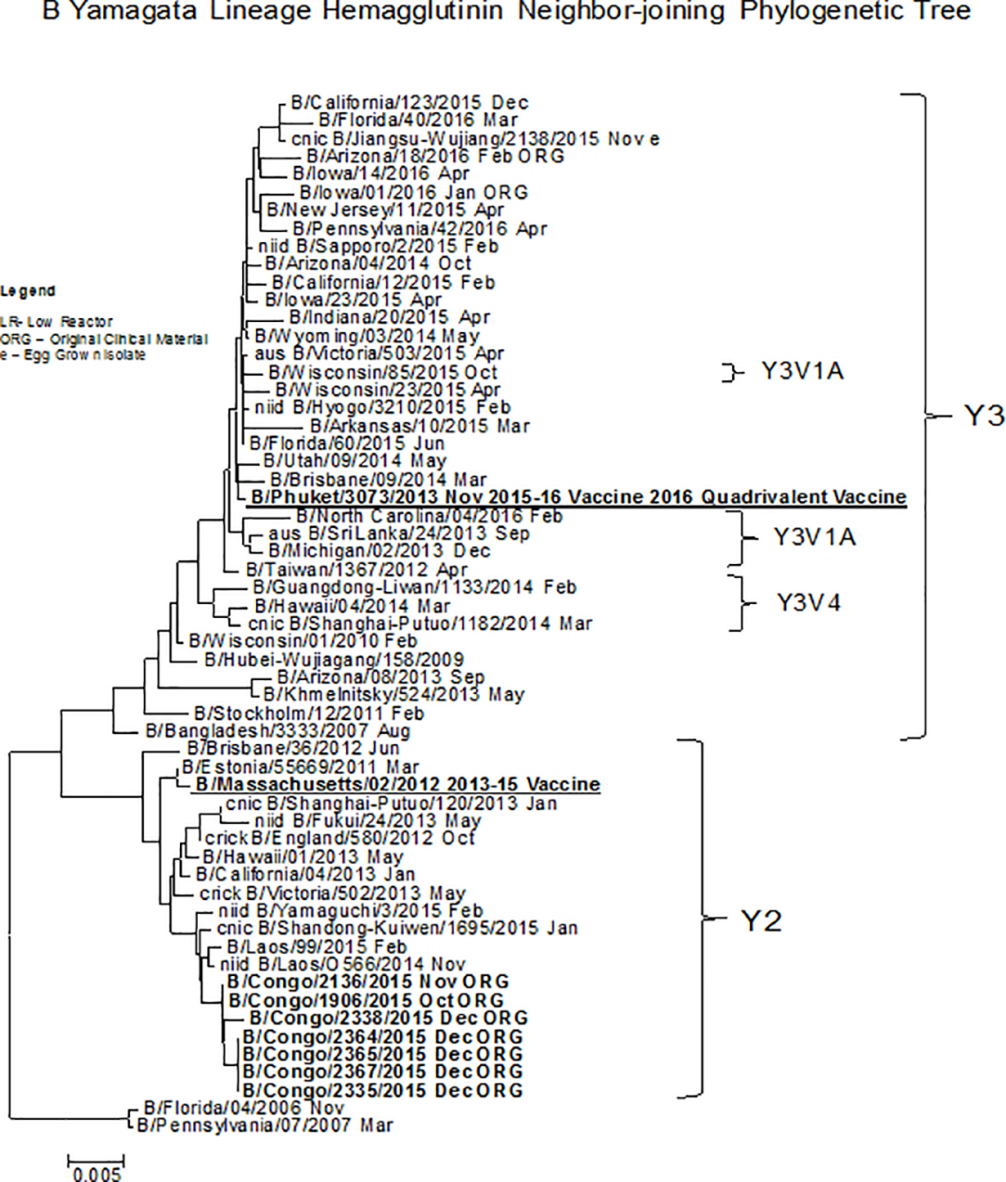
Representative B yamagata Lineage hemagglutinin Neighbor-joining phylogenetic tree of B/Yamagata lineage viruses collected in 2015. The B/Massachusetts/02/2012 (underlined, bold) was used as reference in TREESUB. The consensus HA tree and the transcribed aminoacid substitutions were visualized in fig tree. The scalebar represents the average number of nucleotide substitutions per site. Genetic groups Y3 and Y2 are depicted in the tree. DRC isolates (bold) clustered in the Y2 lineage.

Multiple sequence alignments were carried out for each data set using the CLC Main Workbench 5.7.2 software and phylogenetic trees were constructed using neighbor-joining method [17, 18, 19]. Phylogenetic analysis of the hemagglutinin gene of Flu A (H1N1) viruses in DRC revealed that they were closely related to the 2009 pandemic virus (A/California/07/2009 like) and these viruses fell into genetic group 6B (Fig 3). On the other hand, the hemagglutinin gene of Flu A (H3N2) tested were phylogenetically related to A/Switzerland/9715293/2013. However, the Influenza B (Yamagata) provided by the DRC during 2015, were phylogenetically similar to B/Massachusetts/02/2012 2013–15 and belonged to genetic subgroup 3 (Fig 4). Analysis of susceptibility to the neuraminidase inhibitors exhibited normal sensitive to all antiviral drugs tested (oseltamivir, zanamivir, peramivir and laninamivir.) Table 2.

**Table 2:** Influenza type, sub type and IC 50 values.

LABID^x^	Flu type and sub type	IC_50_^xx^ (fold difference^xxx^)			
		Oseltamivir	Zanamivir	Peramivir	Laninamivir
15GP0026	Type A/ H3N2	0.17 (3)	0.26 (1)	0.09 (1)	0.32 (1)
15GP0001	Type A/ H3N2	0.16 (1)	0.28 (1)	0.09 (1)	0.36 (1)
15GP0002	Type A/ H3N2	0.17 (1)	0.27 (1)	0.08 (1)	0.3 (1)
15GP0004	Type A/ H3N2	0.13 (1)	0.24 (1)	0.08 (1)	0.28 (1)
15GP0008	Type A/ H3N2	0.15 (1)	0.28 (1)	0.09 (1)	0.32 (1)
15GP0015	Type A/ H3N2	0.14 (1)	0.25 (1)	0.1 (1)	0.4 (1)
15 GP 1857	Type A/ H1pdm09	0.16 (1)	0.23 (1)	0.06 (1)	0.19 (1)
15 GP 1942	Type A/ H1pdm09	0.2 (1)	0.21 (1)	0.06 (1)	0.17 (1)
15 GP 1966	Type A/ H1pdm09	0.18 (1)	0.24 (1)	0.07 (1)	0.17 (1)
15 GP 1970	Type A/ H1pdm09	0.16 (1)	0.18 (1)	0.06 (1)	0.24 (1)
15 GP 1973	Type A/ H1pdm09	0.2 (1)	0.2 (1)	0.07 (1)	0.22 (1)
15 GP 2091	Type A/ H1pdm09	0.17 (1)	0.16 (1)	0.06 (1)	0.17 (1)
15 GP 1906	Type B/ Yamagata	10.32 (1)	0.65 (1)	0.3 (1)	1.05 (1)
15 GP 2136	Type B/ Yamagata	6.92 (1)	0.5 (1)	0.33 (1)	0.82 (1)
15 GP 2335	Type B/ Yamagata	8.25 (1)	0.48 (1)	0.25 (1)	0.94 (1)
15 GP 2338	Type B/ Yamagata	8.88 (1)	0.57 (1)	0.33 (1)	1.11 (1)
15 GP 2364	Type B/ Yamagata	12.95 (2)	0.46 (1)	0.3 (1)	0.88 (1)
15 GP 2365	Type B/ Yamagata	7.61 (1)	0.44 (1)	0.24 (1)	0.83(1)
15 GP 2367	Type B/ Yamagata	11.15 (1)	0.46 (1)	0.28 (1)	0.9 (1)

^x^ = Laboratory number (identification)

^xx^ = Generated in fluorescent NI assay

^xxx^ = Compared with the mean IC_50_ for the drug by subtype for influenza viruses A, and by antigenic lineage for type B viruses

## Discussion

Data from this study demonstrated that both Flu A (subtypes H3N2 and H1N1) and B (subtype yamagata) viruses co-circulated in the human population in DRC during 2015. Regarding Flu A, the genotype H3N2 was only predominant in January and February while H1N1 started to be predominant from March and covered the rest of the year 2015. The reason of the short- period spread of H3N2 over the country remain unknown but the most important is that vaccines were made for both of them (H1N1 and H3N2). Contrary to the Flu A profil, only one lineage was present. Indeed, only the B/Yamagata lineage was circulated in DRC from January to December 2015. Similarly, previous studies reported co-circulation of both types of influenza virus in many African countries, including Kenya, Senegal, South Africa, Tunisia and Uganda [20, 21]. Indeed, Flu A (H1N1) pdm09 viruses have continued to circulate worldwide since their emergence in 2009. Moreover, Flu A (H1N1) pdm09 outbreaks closely related to A/California/07/2009-like strains were reported in Asia, Europe, North America and several countries from Africa and Central or South America [22]. Thus, the A/California/07/2009-like virus has been the WHO recommended A(H1N1)pdm09 vaccine component since 2009, including vaccines for the 2016 influenza season in the Southern Hemisphere (SH) and the 2016–2017 influenza season in the Northern Hemisphere (NH) [22].

Genetic characterization revealed that the majority of Flu A(H3N2) viruses tested, were phylogenetically related to A/Switzerland/9715293/2013, the virus which was recommended by the WHO as the Flu A(H3N2) component for the 2015 SH as well as the 2015–2016 NH vaccine formulations [22]. Previous studies reported that both Flu B (B/Victoria/2/87 and B/Yamagata/16/88) lineages have continued to co-circulate, with B/Victoria-lineage viruses predominating in many countries [23, 24, 25]. However, our findings revealed that all Flu B were B/Yamagata and phylogenetically similar to B/Phuket/3073/2013, the second Flu B component of quadrivalent vaccines for the 2016 SH and 2016–2017 NH influenza seasons. The first quarter of the year 2015 was dominated with Flu A (H3N2) co circulating with some Flu B, from March to December 2015, Flu B continued to co circulate with Flu A H1N1pdm which reached its peak in November 2015 (Fig 2).

The emergence of resistance activity to antiviral drug was observed among Cameroonian A(H1N1) isolates in early 2008 and in other African countries [26, 27], while the analyses of of antiviral resistance by enzyme inhibition assay done in this study showed that the DRC isolates were highly sensitive to all antiviral drugs.

It is clear that DRC is one of the biggest African country. Unfortunately, the influenza surveillance system was not able to cover the whole country. Indeed, only 5 out of 11 provinces were selected for implementing influenza surveillance and this could not give the real picture of all Influenza strains circulating in DRC, which considered as a limitation of the data that we could obtain. Results from this study showed that no resistance to antiviral drugs have been detected in DRC samples and this could be because influenza antiviral drugs have never been used extensively in DRC.

## Conclusion

This study documented the circulation of Flu A (H3N2 and H1N1) and Flu B (Yagamata) in five provinces under influenza surveillance. However, the influenza situation in other provinces remains largely unknown. So, there is a need to extend the surveillance in the remaining provinces for a better control and knowledge of circulating influenza strains.

## Supporting information

S1 File Flu dataset.xlsx. (XLSX)

## References

[1] Gessner BD, Shindo N, Briand S. Seasonal influenza epidemiology in sub-Saharan Africa: a systematic review. Lancet Infect Dis. 2011, 11(3):223–35. 10.1016/S1473-3099(11)70008-1 21371656

[2] Kamps BS, Hoffmann C, Preiser J Influenza report on Virology of Human Influenza. Available at www.influenzareport.com/ir/virol.htm. Accessed 17th March 2013. 2006

[3] McAnerney JM, Cohen C, Moyes J, Besselaar TG, Buys A, Schoub BD, et al Twenty-five years of outpatient influenza surveillance in South Africa, 1984–2008. J Infect Dis. 2012, 206 (Suppl 1): S153–S158. 10.1093 23169963 10.1093/infdis/jis575

[4] Barakat A, Ihazmad H, Benkaroum S, Cherkaoui I, Benmamoun A, Youbi M, et alEl (2011): Influenza surveillance among outpatients and inpatients in Morocco, 1996–2009. PLoS One. 2011, 6: e24579– 10.1371/journal.pone.0024579 21931764 PMC3169614

[5] Muyembe Tamfum JJ, Nkwembe E, Bi Shamamba SK, Bankoshi F, Ilunga BK, Katz KA, et al Sentinel surveillance for influenza-like illness, severe acute respiratory illness, and laboratory-confirmed influenza in Kinshasa, Democratic Republic of Congo, 2009–2011. J Infect Dis. 2012, 206 Suppl 1:S36–40. 10.1093/infdis/jis537 23169969

[6] Katz MA, Lebo E, Emukule G, Njuguna HN, Aura B, Cosmas L, et al Epidemiology, seasonality, and burden of influenza and influenza-like illness in urban and rural Kenya, 2007–2010. J Infect Dis. 2012, 206 (Suppl 1): S53–S60. 10.1093/infdis/jis530 23169973

[7] Theo A, Liwewe M, Ndumba I, Mupila Z, Tambatamba B, Mutemba C, et al (2012) Influenza Surveillance in Zambia, 2008–2009. J Infect Dis. 2012, 206: S173–S177. 10.1093/infdis/jis599 23169966

[8] Dawood FS, Iuliano AD, Reed C, Meltzer MI, Shay DK, Cheng PY, et al Estimated global mortality associated with the first 12 months of 2009 pandemic influenza A H1N1 virus circulation: a modelling study. Lancet Infect Dis. 2012, 12: 687–695. 10.1016/S1473-3099(12)70121-4 22738893

[9] Nair H, Brooks WA, Katz M, Roca A, Berkley JA, Madhi SA, et al Global burden of respiratory infections due to seasonal influenza in young children: a systematic review and meta-analysis. Lancet. 2011, 378: 1917–1930. 10.1016/S0140-6736(11)61051-9 22078723

[10] Hilleman MR. Realities and enigmas of human viral influenza: pathogenesis, epidemiology and control. Vaccine. 2002, 20 (25–26): 3068–3087. 10.1016/S0264-410X(02)00254-2 12163258

[11] WHO. Recommended composition of influenza virus vaccines for use in the 2008–2009 influenza season. Wkly Epidemiol Rec. 2008, 83 (9): 81–87. 18309579

[12] WHO. Recommended composition of influenza virus vaccines for use in the 2009 southern hemisphere influenza season. Wkly Epidemiol Rec. 2008, 83 (41): 366–372. 18846716

[13] Potier M, Mameli L, Belisle M, Dallaire L, Melancon SB. Fluorometric assay of neuraminidase with a sodium (4-methylumbelliferyl-alpha-D-N-acetylneuraminate) substrate. Anal Biochem. 1979, 94: 287–296. 10.1016/0003-2697(79)90362-2 464297

[14] Wetherall N, Trivedi T, Zeller J, Hodges-Savola C, McKimm-Breschkin J, Zambon M, Hayden F. Evaluation of Neuraminidase Enzyme Assays Using Different Substrates To Measure Susceptibility of Influenza Virus Clinical Isolates to Neuraminidase Inhibitors: Report of the Neuraminidase Inhibitor Susceptibility Network. J Clin Microbiol. 2003, 41: 742–750. 10.1128/JCM.41.2.742-750.2003 12574276 PMC149673

[15] Zhou B, Wentworth D Influenza A virus molecular virology techniques. Methods Mol. Biol. 2012, 865: 175–192. 10.1007/978-1-61779-621-0_11 22528160

[16] Shepard SS, Meno S, Bahl J, Wilson MM, Barnes J, Neuhaus E. Viral deep Sequencing needs an adaptive approach: IRMA, the iterative refinement meta-assembler. BMC Genomics. 2016, 17: 708 10.1186/s12864-016-3030-6 27595578 PMC5011931

[17] Saitou N, and Nei M. The neighbor-joining method: A new method for reconstructing phylogenetic trees. Molecular Biology and Evolution 1987, 4(4), 406–425 10.1093/oxfordjournals.molbev.a040454 3447015

[18] Gascuel O. BIONJ: An improved version of the NJ algorithm based on a simple model of sequence data. Molecular Biology and Evolution 1997, 14 685–695. 10.1093/oxfordjournals.molbev.a025808 9254330

[19] Studier JA, Keppler KJ (. A note on the neighbor-joining algorithm of Saitou and Nei. Molecular Biology and Evolution 1988, 5 *(*6*)* 729–731. 10.1093/oxfordjournals.molbev.a040527 3221794

[20] WHO. Recommended composition of influenza virus vaccines for use in the 2008–2009 influenza season. Wkly Epidemiol Rec.2008, 83 (9): 81–87.Google Scholar 18309579

[21] WHO. Recommended composition of influenza virus vaccines for use in the 2009 southern hemisphere influenza season. Wkly Epidemiol Rec. 2008, 83 (41): 366–372 18846716

[22] Influenza Division report, Center for Disease Control and Prevention 2016 (Altanta-USA).

[23] Hirve S, Newman LP, Paget J, Azziz-Baumgartner E, Fitzner J, Bha N, et al Influenza seasonality in the tropics and subtropics when to vaccinate. Plos One. 2016, 11(4). 10.1371/journal.pone.0153003 PMC4847850 27119988

[24] Beckett CG, Kasasih H, Ma’roef C, Listiyaningsih E, Elyazar IR, Wuryadi S, et al Influenza surveillance in Indonesia. 1999–2003. Clin. Infect Dis. 2004, 39: 443–449. 10.1086/422314 15356802

[25] Gachara G, Ngeranwa J, Magara JM, Simwa JM, Wango PW, Lifumo SM, et al influenza virus strain in Nairobi, Kenya. J. clin Virol. 2006, 35(1): 117–118. 10.1016/j.jcv.2005.10.004 16309952

[26] Besselaar TG, Naidoo D, Buys A, Gregory V, McAnerney JM, Manamela JM, et al Widespread oseltamivir resistance in influenza A viruses (H1N1), South Africa. Emerg Infect Dis. 2008, 14 (11): 1809–1810. 10.3201/eid1411.080958 18976580 PMC2630761

[27] Njouom R, Sadeuh Mba SA, Noah Noah D, Gregory V, Collins P, Cappy P, et al Hay Circulation of human influenza viruses and emergence of Oseltamivir-resistant A(H1N1) viruses in Cameroon, Central Africa. BMC Infectious Diseases 2010, 10:56 10.1186/1471-2334-10-56 20205961 PMC2838889

